# Structural insight into piezo-solvatochromism of Reichardt’s dye

**DOI:** 10.1107/S2052252524004603

**Published:** 2024-06-04

**Authors:** Szymon Sobczak, Andrzej Katrusiak

**Affiliations:** a Adam Mickiewicz University, ul. Uniwersytetu Poznanskiego 8, Poznań61-612, Poland; Formby, Liverpool, United Kingdom

**Keywords:** solvatomorphism, piezosolvatochromism, high-pressure crystallization, solvation

## Abstract

To date, accurate modelling of the solvation process is challenging, often over-simplifying the solvent–solute interactions. The interplay between the molecular arrangement associated with the solvation process and crystal nucleation has been investigated by analysis of the piezo-solvatochromic behaviour of Reichardt’s dye, ET(1), in methanol, ethanol and acetone under high pressure. High-pressure single-crystal X-ray diffraction and UV–Vis spectroscopy reveal the impact of solute–solvent interactions on the optical properties of ET(1). The study underscores the intricate relationship between solvent properties, molecular conformation and crystal packing. The connection between liquid and solid phases emphasizes the capabilities of high-pressure methods for expanding the field of crystal engineering. The high-pressure environment allowed the determination of the crystal structures reported here that are built from organic molecules fourfold solvated with ethanol or methanol: ET(1)·4CH_3_OH and ET(1)·4C_2_H_5_OH·H_2_O. The observed piezo-solvatochromic effects highlight the potential of ET(1) in nonlinear optoelectronics and expand the application of solvatochromic chemical indicators to pressure sensors.

## Abstract 2

This work employs the preference of compounds to crystallize at high-pressure in the form of solvates to explore the solvation process and piezo-solvatochromic effects of Reichardt’s dye, ET(1). The solute–solvent interactions of this dye affect the optical properties of the dye in various solvents as shown by X-ray diffraction and UV–Vis spectroscopy, revealing applications in nonlinear optoelectronics and molecular pressure sensor development.

## 1. Introduction

Molecular interactions, especially those between solute and solvent molecules, have been the subject of intense scientific investigations for decades (Henkel *et al.*, 2018 ▸; Buncel & Stairs, 2015 ▸; Walker *et al.*, 1992 ▸; Mabesoone *et al.*, 2020 ▸; Laurence *et al.*, 1994 ▸). A particularly intriguing manifestation of these interactions is solvatochromism (Nigam & Rutan, 2001 ▸; Marini *et al.*, 2010 ▸; Bamfield & Hutchings, 2018 ▸), *i.e.* the dependence of absorption of a dye solution on the liquid environment (Reichardt & Welton, 2010 ▸; Machado *et al.*, 2014 ▸; Reichardt, 1994 ▸). This phenomenon, manifested as a colour change, not only provides a visual representation of dye–solvent interactions, but also offers a deep insight into the underlying forces (Plenert *et al.*, 2021 ▸; Spange *et al.*, 2022 ▸) such as hydrogen bonds, dipole–dipole and van der Waals interactions that play a pivotal role for the physicochemical properties of dye solutions (Reichardt, 2004 ▸). However, no structural information about the aggregation modes of the dye and solvent molecules, which could be directly connected with the solvatochromic effects, is available. For the first time, we have employed the techniques of high-pressure crystallization, *in situ*X-ray diffraction and UV–Vis spectroscopy to fill this gap in understanding solvatochromism. It is known that high pressure increases the preference for the formation of solvates, which are often unstable under atmospheric pressure (Bhardwaj *et al.*, 2019 ▸; Boldyreva, 2007 ▸; Fabbiani *et al.*, 2004 ▸, 2010 ▸; Fabbiani & Pulham, 2006 ▸; Katrusiak, 2019 ▸; Marciniak *et al.*, 2016 ▸; Olejniczak *et al.*, 2016 ▸; Oswald *et al.*, 2009 ▸; Safari *et al.*, 2020 ▸; Tomkowiak *et al.*, 2013 ▸; Tumanov *et al.*, 2010 ▸). Our results unveil the interesting interplay between solvent polarity and molecular interactions relevant to various fields of chemistry. The practical aspects of this knowledge involve designing new compounds, chemical synthesis as well as materials science and biochemistry (Reichardt & Welton, 2010 ▸; Buncel & Stairs, 2015 ▸), for example, for developing sensors and drugs (Reichardt, 2004 ▸; Lee *et al.*, 2013 ▸; Klymchenko, 2017 ▸). The solvatochromic effects observed in organic compounds, attributed to their high molecular hyperpolarizability, have spurred interest in their potential applications in nonlinear optoelectronics (Mairesse *et al.*, 2023 ▸; Nie, 1993 ▸; Zyss & Ledoux, 1994 ▸; Champagne & Bishop, 2003 ▸).

**Figure 1:**
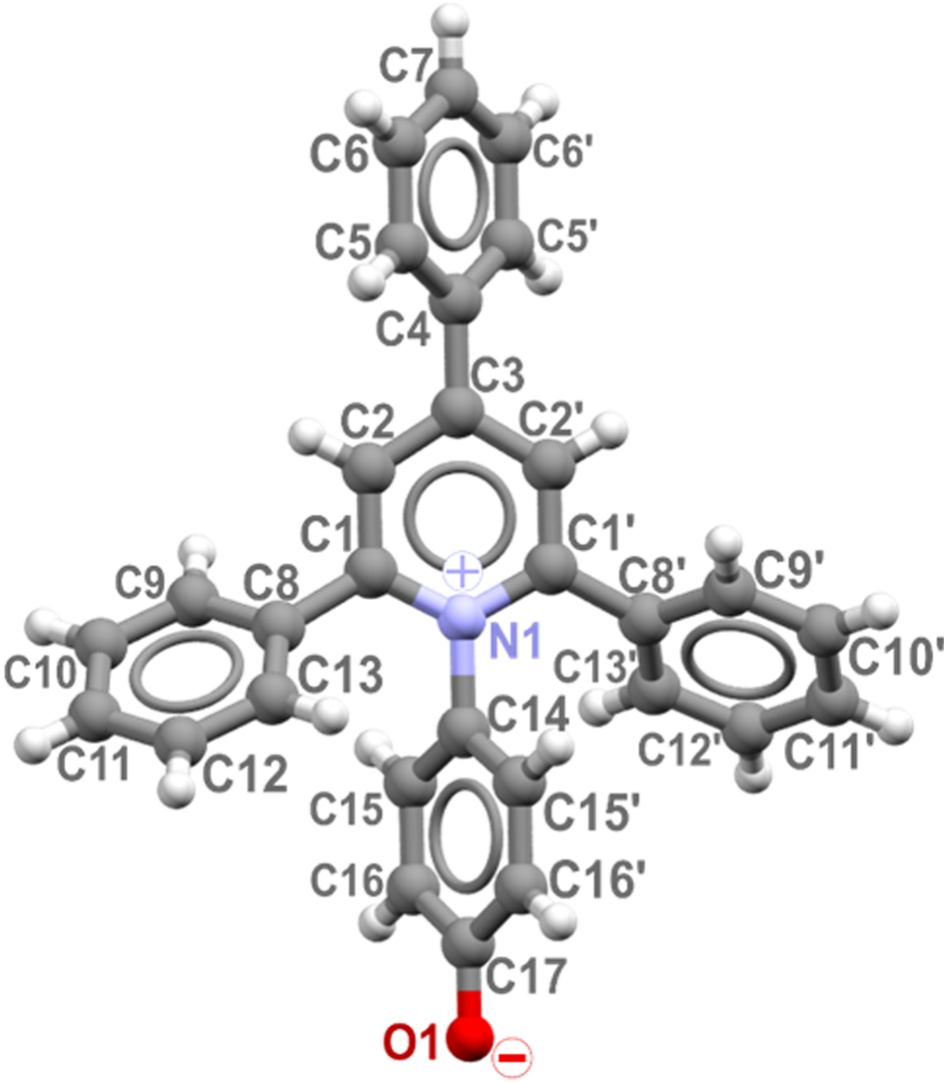
Zwitterion of ET(1) and its atomic labels.

A group of pyridinium *N*-betaine organic dyes, often referred to as Reichardt’s dyes, is particularly sensitive to changes in the solvent environment and thus used to probe solvent–solute interactions (Reichardt, 1994 ▸; Machado *et al.*, 2014 ▸; Dimroth *et al.*, 1963 ▸). The molecules of Reichardt’s dyes comprise an extended conjugated and polarizable system, with a large permanent dipole moment (Budzák *et al.*, 2017 ▸; Walker *et al.*, 1992 ▸). Additionally, the oxygen atom on the phenolate moiety is highly basic and prone to form of hydrogen bonds with solvent molecules (Spange & Weiß, 2023 ▸; Spange *et al.*, 2022 ▸; Plenert *et al.*, 2021 ▸). The simplest representative among the *N*-betaine dyes is 4-(2,4,6-tri­phenyl-1-pyridinio)phenolate, herein referred to as ET(1), shown in Fig. 1 ▸.

In order to explore the structure–property relationships of solvatochromic effects, we applied high-pressure *in situ* crystallization to obtain and stabilize the ET(1) methanol and ethanol solvates, which have been investigated by high-pressure single-crystal X-ray diffraction. Through this comprehensive investigation, we aspire to deepen our understanding of the solvation and solvatomorphism phenomena, as well as establish high-pressure crystallization combined with high-pressure spectroscopy as the stage for future research and applications in this captivating domain.

## 2. Experimental

**Figure 2:**
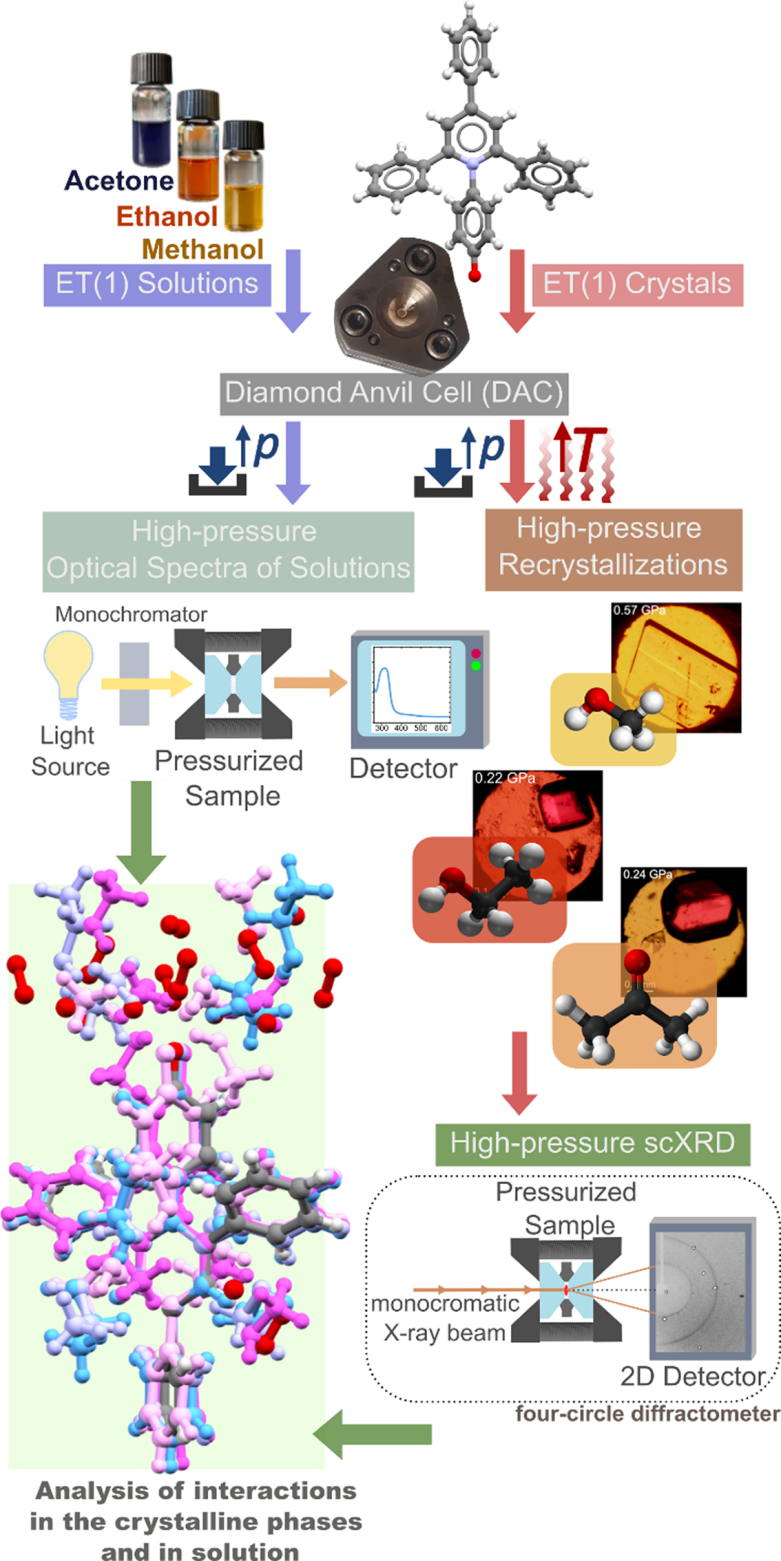
Schematic overview of experimental procedures.

The most common synthetic approach to produce pyridinium *N*-phenolates is the procedure established by Dimroth *et al.* (1963 ▸), involving the reaction of a pyrylium salt with an amino­phenol, followed by the reaction of the protonated dye with sodium methoxide or sodium (or potassium) hydroxide. This synthesis yields red single crystals of ET(1) (Dimroth *et al.*, 1963 ▸). The crystals analysed in the high-pressure experiments here were used without any further purification. A graphical scheme of the experimental procedure for the investigation piezo-solvatomorphism is illustrated in Fig. 2 ▸.

### 2.1. High-pressure X-ray diffraction

The *in situ* crystallization of all solvates was conducted using a modified Merrill–Bassett diamond anvil cell (DAC) (Merrill & Bassett, 1974 ▸). For the experiments, a 0.3 mm-thick steel gasket with a 0.45 mm diameter hole was used. The single crystals were grown under isochoric conditions according to the following procedure: (i) red single crystals of ET(1) and a chosen solvent were loaded into the DAC chamber and compressed; (ii) the DAC was heated with a hot-air gun until all but one grain of ET(1) melted; (iii) through the controlled cooling of the DAC to room temperature, a single crystal was grown (Katrusiak, 2019 ▸). The temperature was monitored using an infrared thermometer and the pressure was calibrated with the ruby-fluorescence method before and after each X-ray diffraction analysis with a photon control spectrometer, affording an accuracy of 0.02 GPa (Mao *et al.*, 1986 ▸; Piermarini *et al.*, 1975 ▸).

Data collection and reduction were performed using the *CrysAlisPro* software (Rigaku, 2014 ▸). The crystal structures were solved and refined using *SHELX* (Sheldrick, 2008 ▸, 2015 ▸) within the *Olex2* software (Dolomanov *et al.*, 2009 ▸). Crystallographic data and experimental details are compiled in Table 1 ▸ and Table S1 of the supporting information, and have been deposited in CIF format in the Cambridge Crystallographic Database Centre (CCDC numbers provided). Structural illustrations were generated using *Mercury* (Macrae *et al.*, 2008 ▸).

### 2.2. High-pressure optical absorption

UV–Vis measurements at high pressure were conducted with a Merrill–Bassett DAC equipped with type IIa diamonds, with 0.8 mm-diameter diamond culets. The gaskets were made of 0.1 mm-thick foil with a sparked-eroded hole 0.45 mm in diameter. Solutions of ET(1) (1.1 m*M* concentration) were prepared at room temperature and pressure and loaded into the DAC along with a small ruby chip, without any additional pressure-transmitting medium. High-pressure UV–Vis spectra were recorded using a Jasco V-770 spectrophotometer, adapted for the DAC. Absorbance measurements were carried out at a scan rate of 200 nm min^−1^ in the 250–800 nm wavelength range.

## 3. Discussion and results

Solvation is a complex process that can be considered from many perspectives (Reichardt & Welton, 2010 ▸). This phenomenon can be considered on the level of the direct interactions formed between solute and solvent, often referred to as microsolvation (Rahbar & Stein, 2023 ▸). On the other hand, solvation can be viewed from the global perspective, where the solute molecules are stabilized by the general properties of the solvent, then called macrosolvation (Plenert *et al.*, 2021 ▸). Both these approaches describing the solvent–solute environment are closely related, although they fail when strong, direct intermolecular interactions, such as electrostatic, van der Waals forces and hydrogen bonds affect the solvent–cluster conformation in the bulk solvent, which strongly influence the long-range solvent polarization effects (Plenert *et al.*, 2021 ▸).

One of the most common empirical indicators used for determining the magnitude of the solvent–solute interactions is the ET(30) scale, introduced by Reichardt (1994 ▸) based on the solvatochromic response of 2,6-di­phenyl-4-(2,4,6-tri­phenyl­pyridinio)phenolate, often referred to as ET(30). The ET(30) scale is frequently used for measuring the molar electronic transition energies of pyridinium *N*-phenolate betaine dye in various solvents (Cerón-Carrasco *et al.*, 2014 ▸). Although it was recently postulated that the ET(30) scale does not reflect the solvent ‘polarity’ but rather the global polarity of alcoholic solvents represented by the number of OH groups per volume, *i.e.* hydroxyl-group density (Spange *et al.*, 2022 ▸; Spange & Weiß, 2023 ▸), it is still used by many theoretical and experimental approaches for investigating complicated and entwined solvent–solute interactions.

### 3.1. Solvatochromism of ET(1) at high-pressure

All pyridinium *N*-phenolates demonstrate a typical spectrum with an intense short-wavelength main band around 300 nm, which is hardly influenced by solvents, and a weaker highly solvent-sensitive solvatochromic band or bands between 350 and 800 nm, often referred to as the intramolecular charge-transfer (λ_CT_) absorption band (Dimroth *et al.*, 1963 ▸; Reichardt, 2004 ▸, 1994 ▸; Machado *et al.*, 2014 ▸). The position of the main absorption band (λ_max_) is also frequently associated with the absorbance of the *N*-substituted 2,4,6-tri­phenyl-pyridinium cation, as the band position is preserved even after the addition of an acid to the solution mixture, while the solvatochromic bands disappear completely (Reichardt *et al.*, 2001 ▸). The solvatochromic effect in this group of compounds was postulated to originate from differences between solvation spheres formed around the dye’s highly dipolar electronic ground state and its less dipolar excited state (Reichardt, 1994 ▸). With increasing solvent polarity, the dipolar electronic ground state is more stabilized by the interaction with solvent than the less polar, excited state π–π*. The charge-transfer (CT) band of ET(1), like for other Reichardt’s dyes, is extremely sensitive to the presence of water, especially in nonpolar solvents, to the point that contamination by the crystallization water can significantly alter the position of the CT band (Stadnicka *et al.*, 2002 ▸).

The influence of the external pressure on the solvation process and on the interactions between organic solvents, often used in high-pressure techniques as pressure-transmitting media, remains unsolved and is frequently suggested as a reason behind unexpected phenomena (Zakharov *et al.*, 2016 ▸; Zakharov & Boldyreva, 2019 ▸; Sobczak & Katrusiak, 2019 ▸). The spectroscopic investigation on the solutions of a model ET(30) betaine and 4-(pyridinium-1-yl)phenolate at high-pressure carried out previously by Drickamer’s (Hammack *et al.*, 1989 ▸) and Kelm’s groups (Jouanne *et al.*, 1978 ▸) confirm the expected shift of the CT absorption band to a shorter wavelength (higher frequency) with increasing pressure. This hypsochromic shift called piezo-solvatochromism (Machado *et al.*, 2014 ▸) was postulated as a result of the pressure-supported stabilization of the dye’s zwitterionic ground state. Furthermore, the observed changes in λ_max_ and λ_CT_ have been shown to correlate well with the increase of the dielectric function (ɛ_r_ − 1)/(ɛ_r_ + 2) of the solvent on compression (Hammack *et al.*, 1989 ▸). This indicates that nonspecific solute/solvent interactions primarily govern the piezo-solvatochromic behaviour (Machado *et al.*, 2014 ▸; Reichardt, 1992 ▸), whereas the solvents that can be involved in the formation of strong hydrogen bonds appear to be less sensitive to pressure changes (Reichardt, 1992 ▸).

**Figure 3:**
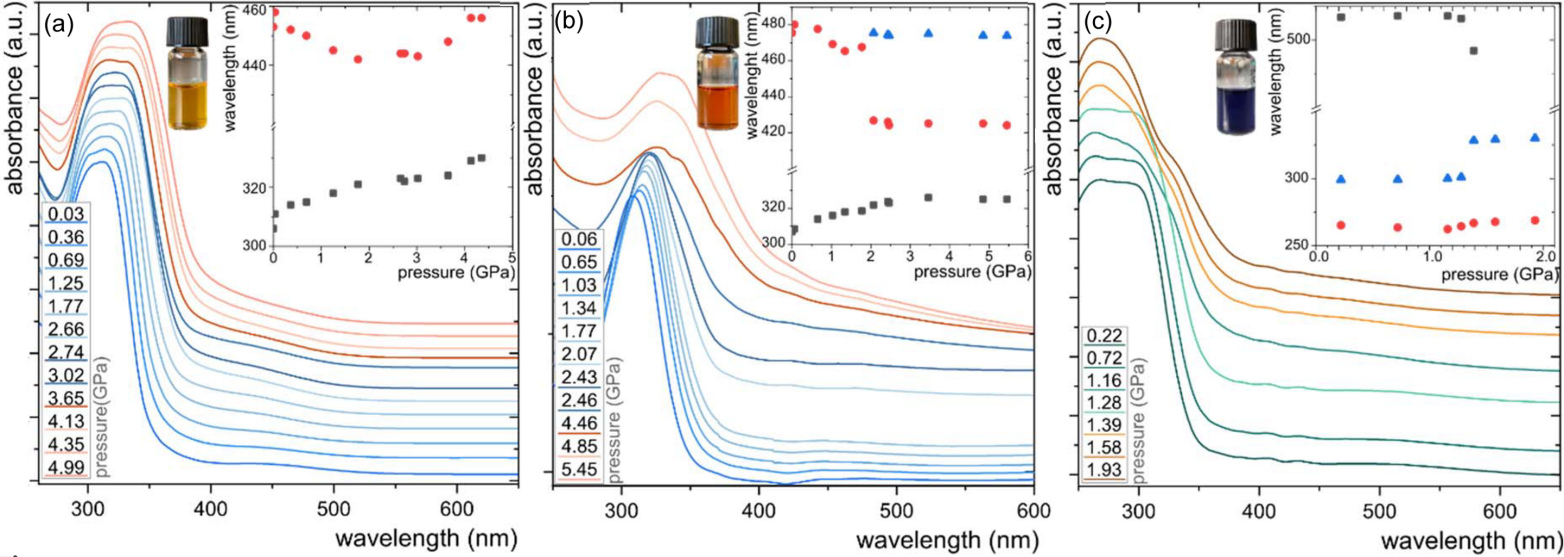
UV–Vis absorption of the ET(1) solution in (*a*) methanol, (*b*) ethanol and (*c*) acetone as a function of pressure. The inset plots show the changes in the maximum peak position with pressure. The photographs of vials with 1.1 m*M* solutions loaded in the DAC illustrate their colour at ambient pressure.

In light of these findings, in order to better understand the solvation process we investigated the UV–Vis spectra at high-pressure of ET(1) solutions in three different polar solvents: methanol, ethanol and acetone (Fig. 3 ▸). The three distinct colours of those mixtures strongly correlate with the hydrogen-bonding capabilities of the solvents; ET(1) dissolved in methanol is yellow, in the ethanol solution it is red and in the acetone solution it is blue, as shown in Fig. 3 ▸ (Dimroth *et al.*, 1963 ▸; Stadnicka *et al.*, 2002 ▸). These ET(1) solutions were loaded into a DAC and their spectra were measured as a function of high pressure.

The UV–Vis spectra of a methanol solution collected at 0.03 GPa show λ_max_ at 311 nm and λ_CT_ at 458 nm [Fig. 3 ▸(*a*)]. The results correlate with previously reported values at room temperature and pressure, where λ_max_ was localized at 306 nm and λ_CT_ at 452.8 nm, the small inconsistency in the position of the bands can be related to the large width of peaks and the method chosen for locating the peak centre (Dimroth *et al.*, 1963 ▸; Stadnicka *et al.*, 2002 ▸). When pressure is increased to 1.77 GPa, λ_max_ blue-shifts linearly to 321 nm at a rate of Δλ_max_ = 5.75 nm GPa^−1^. At the same time, the rate of hypsochromic shift of the CT band is Δλ_CT_ = 8.6 nm GPa^−1^. Further compression of the solution leads to the crystallization of the solution occurring at about 0.07 GPa above the crystallization pressure of pure methanol at *p*_c_ = 3.5 GPa according to Allan *et al.* (1998 ▸). Interestingly, on approaching methanol crystallization, there are anomalous changes in the pressure dependence of the absorbance spectra. While the constant increase of λ_max_ reflects the growing strain and increased potential energy (*E*_p_) of the ET(1) molecule, the hypsochromic trend for λ_CT_ is reversed above 3 GPa, where it becomes bathochromic. This unprecedented bathochromic shift to 456 nm above 4.33 GPa corresponds to the solvatochromic band position at around 0.3 GPa. This U-turn shift clearly shows that, while the zwitterionic form of the ET(1) is stabilized in the solvation sphere, the interactions formed with the solvent molecules are significantly altered above the hydro­static limit of the solvent.

Both characteristic bands λ_max_ = 308.5 and λ_CT_ = 480 nm measured at 0.06 GPa for the red-orange ethanol solution coincide with that reported at room temperature and pressure of λ_max_ = 307 nm and λ_CT_ = 478 nm (Dimroth *et al.*, 1963 ▸; Stadnicka *et al.*, 2002 ▸). Note that, although the wide and asymmetric shape of the CT band significantly hindered the accurate determination of the λ_CT_ maximum peak, the trend is clear [Fig. 3 ▸(*b*)]. On compression to 2.07 GPa, the monotonic bathochromic shift of λ_max_ to 321.8 nm takes place. The shift rate Δλ_max_ = 6.75 nm GPa^−1^ indicates that the potential energy of molecule ET(1) compressed in ethanol is more strongly affected than in methanol. At the same time, the λ_CT_ shift is intriguing. Up to 0.65 GPa, the pressure shifts the CT band towards shorter wavelengths (Δλ_CT_ = 4.07 nm GPa^−1^), but at higher pressure the changes are fourfold more pronounced, and at 1.34 GPa the Δλ_CT_ reaches 17.8 nm GPa^−1^. Interestingly, above 2 GPa the observed bathochromic trend is reversed and at 2.07 GPa the CT band splits into λ_CT1_ at 426.7 nm and λ_CT2_ at 475.5 nm [Fig. 3 ▸(*b*)]. These unexpected changes in absorbance spectra precede the solidification of the solution at 2.32 GPa, about 0.5 GPa above the freezing pressure of pure ethanol (*p*_c_ = 1.8 GPa) (Anderson *et al.*, 1998 ▸). Both split CT bands are hardly affected; further compression to 5.45 GPa red-shifts λ_CT1_ to 424 nm and λ_CT2_ to 474 nm. The non-hydro­static compression of ET(1) dissolved in ethanol shifts λ_max_ at 5.45 GPa to 325 nm.

The dark blue colour of the acetone solution is distinct from those of the methanol and ethanol solutions [Fig. 3 ▸(*c*)]. At 0.22 GPa the ET(1) absorbance band has a wide, rounded shape, and thus we decided to deconvolute this signal into two peaks: λ_max1_ at 265.2 and λ_max2_ at 298.9 nm. At this pressure, λ_CT_ can be located at 517 nm, which is significantly lower than the λ_CT_ = 561 nm reported previously at 0.1 MPa. This difference exceeds the expected pressure effect, which can be due to the shape of the CT band and some water contamination, proven to shift the λ_CT_ position by 2000 cm^−1^. The compression does not shift the absorption bands as strongly as the protic solvents and λ_CT_ at 1.28 GPa is 516 nm. Although the bathochromic change of λ_CT_ is visible, the rate of pressure-induced shift is low, with Δλ_CT_ = 0.92 nm GPa^−1^. The compression to 1.28 GPa also changes the λ_max1_ and λ_max2_ positions: λ_max1_ becomes blue-shifted to 264.4 nm while λ_max2_ is red-shifted to 301 nm. These opposite effects suggest that only λ_max1_ can be directly associated with the *E*_p_ increase of the ET(1) molecule, whereas for λ_max2_ some contributions of the solvation sphere are apparent. Above 1.39 GPa, the strong changes in shape and peak position are visible, which precede the observed solidification of the mixture at 1.78 GPa, 0.28 GPa higher than pure acetone (Allan *et al.*, 1999 ▸). The crystallization of acetone leads to extinction of the weak CT band, but is accompanied by the abrupt change in the λ_max2_ position. At 1.39 GPa, λ_max1_ = 266.8 nm while λ_max2_ shifts about 27 nm and reaches λ_max2_ = 328 nm. Compression to 1.93 GPa further shifts the bands to 269 and 330 nm for λ_max1_ and λ_max2_, respectively.

### 3.2. High-pressure solvates of ET(1)

The crystal structure of ET(1) at room temperature was first reported by Stadnicka *et al.* (2002 ▸), who revealed significant instability of the crystals. The quality of the red needle-like crystals of ET(1) depends on the temperature and humidity (Stadnicka *et al.*, 2002 ▸). This degradation is manifested as a colour change from red to blue. The X-ray diffraction data, collected for the red crystals coated with silicone oil under ambient conditions, show that ET(1) zwitterions crystallize in the orthorhombic space group *C*222_1_, forming a hydrate with a non-stoichiometric amount of water (5.78 molecules per structural unit). In this crystal structure, the torsion angle between the phenolate and the pyridinium ring is equal to 60.0 (2)° (Stadnicka *et al.*, 2002 ▸). Such a conformation was also reported for protonated ET(1) in bi­phenyl-4-sulfonic and 4-amino­benzene­sulfonic salts (Wojtas *et al.*, 2004 ▸).

Our attempts to obtain single crystals of ET(1) from different solvents at room temperature confirmed the previously reported challenges (Stadnicka *et al.*, 2002 ▸), which we tackled by stabilizing the crystal phases under the strictly controlled thermodynamic conditions in the DAC. This prompted us to perform isochoric recrystallizations in a DAC (Katrusiak, 2008 ▸, 2019 ▸) aimed at the structural determination of ET(1) molecules in their solvation environment in the solid state, analogous to the solvatochromic effects observed in the solutions of methanol, ethanol and acetone for which we measured the absorption spectra under pressure.

**Figure 4:**
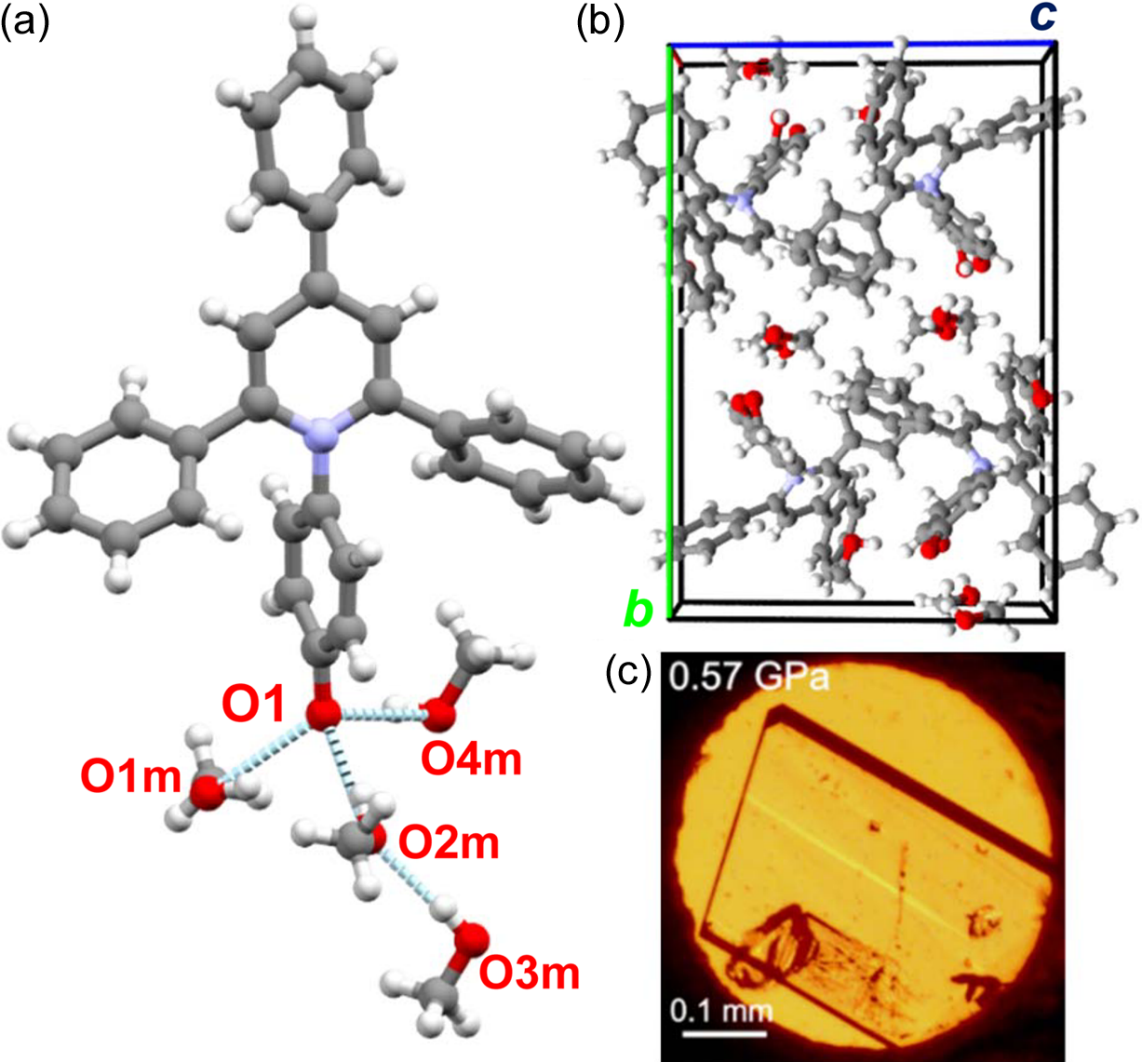
(*a*) Hydrogen bonds between ET(1) and four methanol molecules in the crystal structure of ET(1)·4CH_3_OH at 0.57 GPa. (*b*) Unit cell of ET(1)·4CH_3_OH viewed along crystal direction [100]. (*c*) Single crystal of ET(1)·4CH_3_OH grown in the DAC at 0.57 GPa.

The isochoric crystallization of the ET(1) in methanol at 0.57 GPa and 1.17 GPa yielded yellow single-crystal plates of the ET(1) tetra­methanol solvate, ET(1)·4CH_3_OH. It is remarkable that these crystals and the ET(1) methanol solution are very similar in colour (Fig. 4 ▸), which can be an indication that the solvation spheres are very similar for the solution and the solvate. The ET(1)·4CH_3_OH solvate crystallizes in the monoclinic system with the space group *P*2_1_/*n*. One ET(1) molecule hydrogen bonded to four methanol (CH_3_OH) molecules constitutes the asymmetric unit. At 0.57 GPa, the hydrogen bonds around the carbonyl oxygen O(1) can be represented by O(1)⋯H—O1m, O(1)⋯H—O4m, O(1)⋯H—O2m, in addition to the hydrogen bond between methanol molecules, O2m⋯H—O3m of 1.951 (18) Å. The solvate molecules are positioned on the (101) crystallographic plane, which separates the C—H⋯π bonded ET(1) molecules. There are two C—H⋯π aggregates present in ET(1)·4CH_3_OH: (i) cyclomer of four ET(1) molecules with hydrogen bonds involving the lateral phenyl rings perpendicular to crystal direction [001]; and (ii) a chain of interlocking ET(1) molecules along the [101] direction linked by hydrogen bonds involving phenolate moieties and phenolate rings. The conformation of zwitterions of ET(1) is defined by the rotation of the *N*-phenolate ring, described by the torsion angles C1—N1—C14—C15′ (τ_1_) and C1′—N1—C14—C15 (τ_1_′). Additionally, the conformation of three phenyl substituents is described by the torsion angles C2—C3—C4—C5 (τ_2_), C2′—C3—C4—C5′ (τ2′), C2—C1—C8—C13 (τ_3_), N1—C1—C8—C9 (τ_3_′), C2′—C1′—C8′—C13′ (τ_4_) and N1—C1′—C8′—C9′ (τ_4_′), as illustrated in Fig. 1 ▸ and detailed in Table 1 ▸

At 1.17 GPa, the conformation of zwitterion ET(1) is retained except for minor changes in the torsion angles. The increased pressure tightens the voids around solvent molecules, particularly noticeable along the [010] direction which is *ca* 0.5 Å shorter compared with that at 0.57 GPa. The strong coupling between the pyridinium and phenolate ring in the methanol solvate is best represented by the short N(1)—C(14) and O(1)—C(17) bond distances. The N(1)—C(14) bond decreased from 1.437 (15) Å at 0.57 GPa to 1.41 (3) Å at 1.17 GPa, which is much shorter compared with other crystalline betaine dyes (Baran *et al.*, 2001 ▸; Shekhovtsov *et al.*, 2012 ▸; Schowner *et al.*, 2018 ▸; Wojtas *et al.*, 2006 ▸, 2004 ▸; Kurjatschij *et al.*, 2010 ▸; Pike *et al.*, 2018 ▸). At the same time, the C(17)—O(1) bonds of 1.306 (17) and 1.31 (3) Å at 0.57 and 1.17 GPa, respectively, are longer than those found in other solvates and shorter than the C(17)—O(1)H bond present in the salts of similar dyes. The conformation of ET(1) in this solvate confirms the delocalization of the negative charge at O(1), improving its hydrogen-acceptor capability. As presented in Table 2 ▸, the corresponding torsion angles τ_1_ and τ_1_′ are above 70° and become more open with higher pressure. The conjugation of the π-electrons in the betaine core is shown by the alignment of the apex *p*-phenyl substituent, with respect to the central *N*-phenolate ring, with τ_2_ and τ_2_′ of about 35°. The conformation of other phenyl substituents characterized by τ_3_ and τ_3_′ as well as τ_4_ and τ_4_′ values is strongly affected due to the presence of stacking aggregates and thus they assume a more twisted conformation with torsion angles above 50°.

**Figure 5:**
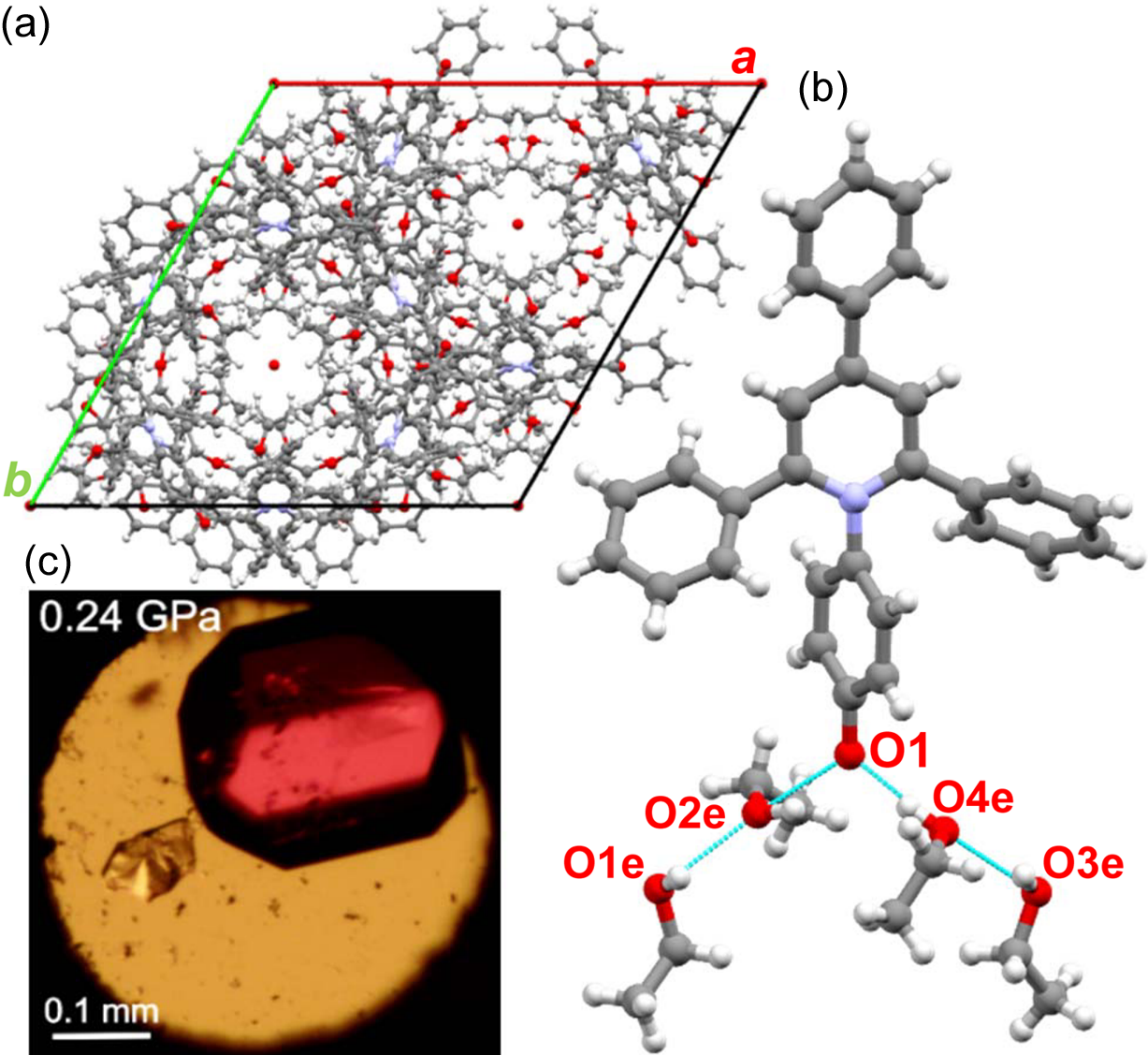
(*a*) Unit cell of ET(1)·4C_2_H_5_OH·H_2_O viewed along the [001] direction. (*b*) Asymmetric part of the unit cell in ET(1)·4C_2_H_5_OH·H_2_O with hydrogen bonds indicated. (*c*) Single crystal grown in the DAC at 0.24 GPa.

The isochoric recrystallization of ET(1) at 0.24 GPa and 0.76 GPa from ethanol (C_2_H_5_OH) solution yielded red single crystals, again similar to the colour of the ET(1) solution in ethanol. The high-pressure X-ray diffraction experiments revealed that we obtained yet another solvate. This three-component compound ET(1)·4C_2_H_5_OH·H_2_O crystallizes in the trigonal system with the space group *R*3*c* (Fig. 5 ▸). We emphasize that, owing to the limitations of the high-pressure technique and the complexity of the structure, the data collected did not allow us to unambiguously confirm the noncentrosymmetric nature of this solvate. However, with the aim of minimizing the deviation of the bond lengths and the size of the thermal vibration ellipsoids as well as the *R*_1_ and *R*_2_ parameters, we chose a symmetry without an inversion centre. The structure of ET(1)·4C_2_H_5_OH·H_2_O is porous, resembling the solvates of ET(30), obtained from vapour diffusion crystallization of di­ethyl ether into solutions of chloro­form, di­chloro­methane, aceto­nitrile and 1-octanol (Pike *et al.*, 2018 ▸). These solvates of ET(30) are also porous with disordered solvent molecules filling the channels extending along the [001] direction [see Fig. 5 ▸(*a*)]; however, all crystallize in the centrosymmetric space group *R*3*c*. The void analysis performed with the probing sphere radius of 1.40 Å (*Olex2*) of ET(1)·4C_2_H_5_OH·H_2_O at 0.24 GPa shows that the channels have a minimum internal diameter of 4 Å and the largest spherical cavity of 7.2 Å across. In this solvate, the ET(1) molecules occupy only about 39.50% of the total crystal volume, which is unexpected as the high pressure usually favours a dense, close molecular packing. What is more, at 0.76 GPa the pores are still not eliminated with the void radius reduced only to 3.4 Å.

It is apparent that the packing arrangement in ET(1)·4C_2_H_5_OH·H_2_O at high pressure originates from the strong hydrogen bonds with guest solvents and intermolecular edge-to-face C—H⋯π interactions formed between lateral phenyl rings, as well as the phenolate moiety and lateral phenyls. Although the ET(1) molecules do not form C—H⋯O bonds with aromatic protons, as was reported for ET(30) solvates (Pike *et al.*, 2018 ▸), the stacking interactions similarly introduce a 120° angle between the *C*_2_ central axis of the molecules, symmetrically related by a twofold rotation about its central core, producing a ‘trigonal node’ and the hexagonal structure. Its framework is supported by hydrogen bonds between the carbonyl atom O(1) and solvent C_2_H_5_OH. The O1e—H⋯O2e—H⋯O(1)⋯H—O4e⋯H—O3e hydrogen bonds in the crystal structure are depicted in Fig. 5 ▸(*b*). The strongly disordered water molecules, located on special positions inside the cavities, do not form strong interactions with ET(1) and ethanol molecules.

In the ET(1)·4C_2_H_5_OH·H_2_O solvate, the ET(1) molecule is more strongly conjugated than in the methanol solvate. This is shown by a flatter conformation of the ET(1) core, specifically connected to torsion angles τ_1_ and τ_1_′ both approximately 60°, and τ_2_ and τ_2_′ both around 30°, as detailed in Table 2 ▸. The flatter structure is more favourable for the π-electron delocalization within the ET(1) core. The conjugation between the rings can be further confirmed by the N(1)—C(14) bond length of 1.45 (2) Å which is not affected as the pressure changes from 0.24 to 0.76 GPa. In contrast, the O(1)—C(17) bond decreases from 1.323 (17) to 1.308 (6) Å, respectively. The alignment of the lateral phenyl substituents, indicated by the pairs of angles τ_3_ and τ_3_′, τ_4_ and τ_4_′, are all around 50°, showing that the ET(1) molecule adopts its conformation to its strong C—H⋯π bonds.

**Figure 6:**
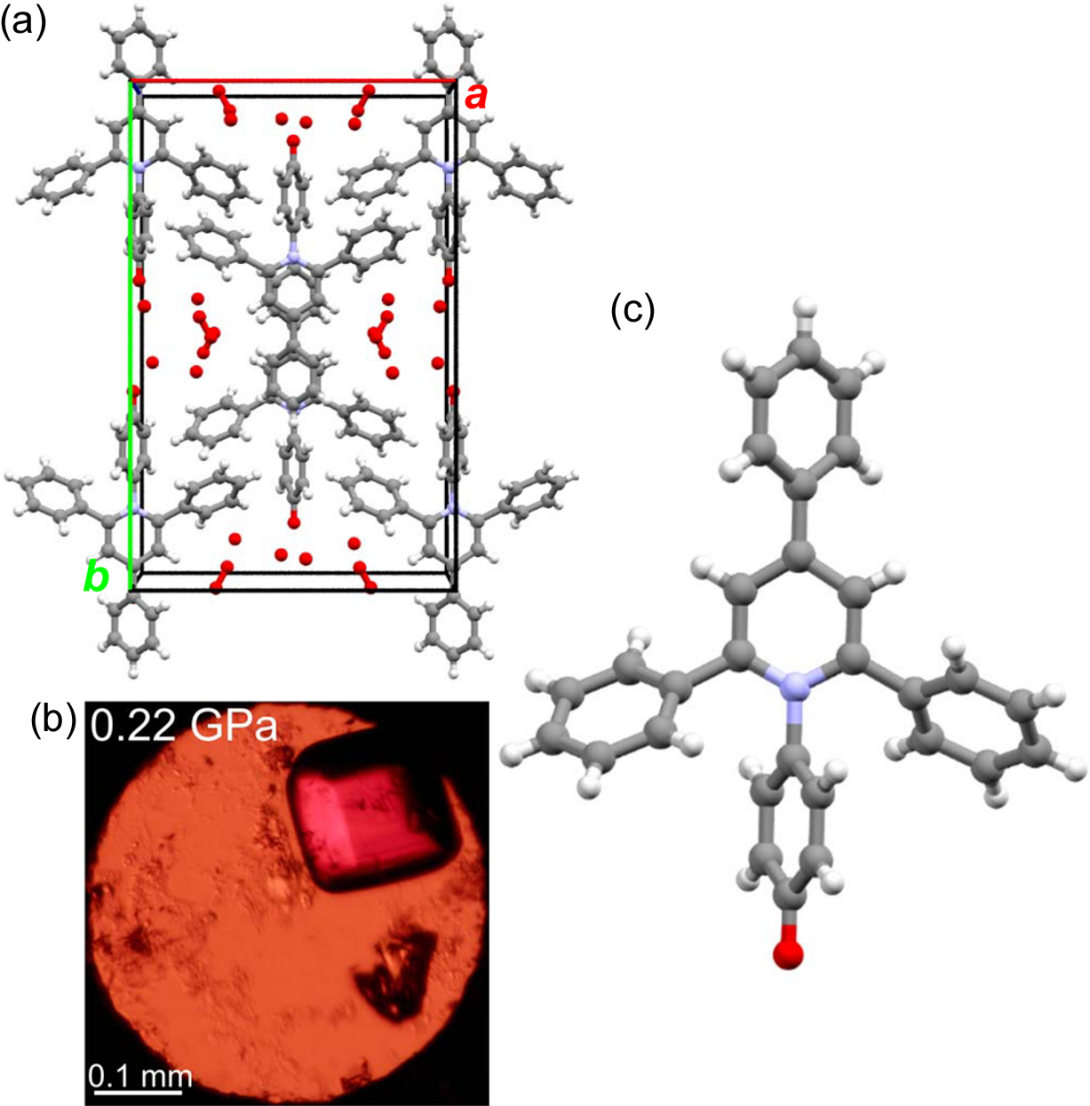
(*a*) ET(1)·6H_2_O crystal structure with water-filled pores along the [001] direction. (*b*) Single crystal of ET(1)·6H_2_O in the DAC at 0.22 GPa. (*c*) The conformation of ET(1) molecule in ET(1)·6H_2_O.

The successful high-pressure crystallization from the acetone solution at 0.22 GPa (see Fig. 6 ▸) yielded another solvate. The single crystal of hexahydrate ET(1)·6H_2_O is isostructural to the previously determined hydrate ET(1)·5.78H_2_O obtained at room temperature and pressure. Both these crystals are orthorhombic with the space group *C*222_1_. The ET(1) molecule in ET(1)·6H_2_O is located on a twofold axis along the *C*_2_ axis of its core. The ET(1) molecules are stacked in a distinct antiparallel mode in columns along the crystal direction [001]. This stacking involves the central pyridinium ring and the core phenyl moiety of the neighbouring molecule related by the symmetry of twofold axis, but the mean planes of these rings are inclined by 24.8 (2)°. Nonetheless, the shortest contacts between atoms C(3) and C(5) of these rings are 3.06 (1) Å long and reassemble pancake bonds. These contacts affect the position of *p*-phenyls, twisting their conformation at 0.22 GPa compared with the ET(1)·5.78H_2_O structure under normal conditions. The disordered water molecules are located along the channels in the [001] crystal direction between the π-stacked columns and do not form any short contacts with the ET(1) molecule. The solvent-accessible channels, including the largest spherical voids of 2.60 Å in radius, occupy 47.3% of the crystal volume. This allows the guest solvent molecules to be easily transported within the crystal, which is manifested by the non-stoichiometric number of guest molecules in the crystals exposed to the room environment. Thus, the higher water content at high pressure, confirmed by slightly larger unit-cell dimensions of the high-pressure hydrate, results from the confined environment inside the DAC, enforcing the guest molecules to remain in their most favoured positions in the crystal structure.

In ET(1)·6H_2_O under high pressure, the molecular structure of ET(1) is more planar, with an enhanced π-electron delocalization across the molecule. This planarity is particularly reflected in torsion angles τ_1_ = 57.1 (3)°, τ_2_ = 49.2 (3)°, τ_3_ = 40.2 (2)° and τ_4_ = 57.0 (3)°, indicating a significantly narrower alignment of the phenyl rings with the central pyridinium ring, facilitating a strong conjugation within the ET(1) core. Similarly, the C(17)—O(1) and N(1)—C(14) bond lengths are 1.252 and 1.499 Å, respectively, which is much shorter than those observed under ambient conditions and in ET(1)·6H_2_O.

**Figure 7:**
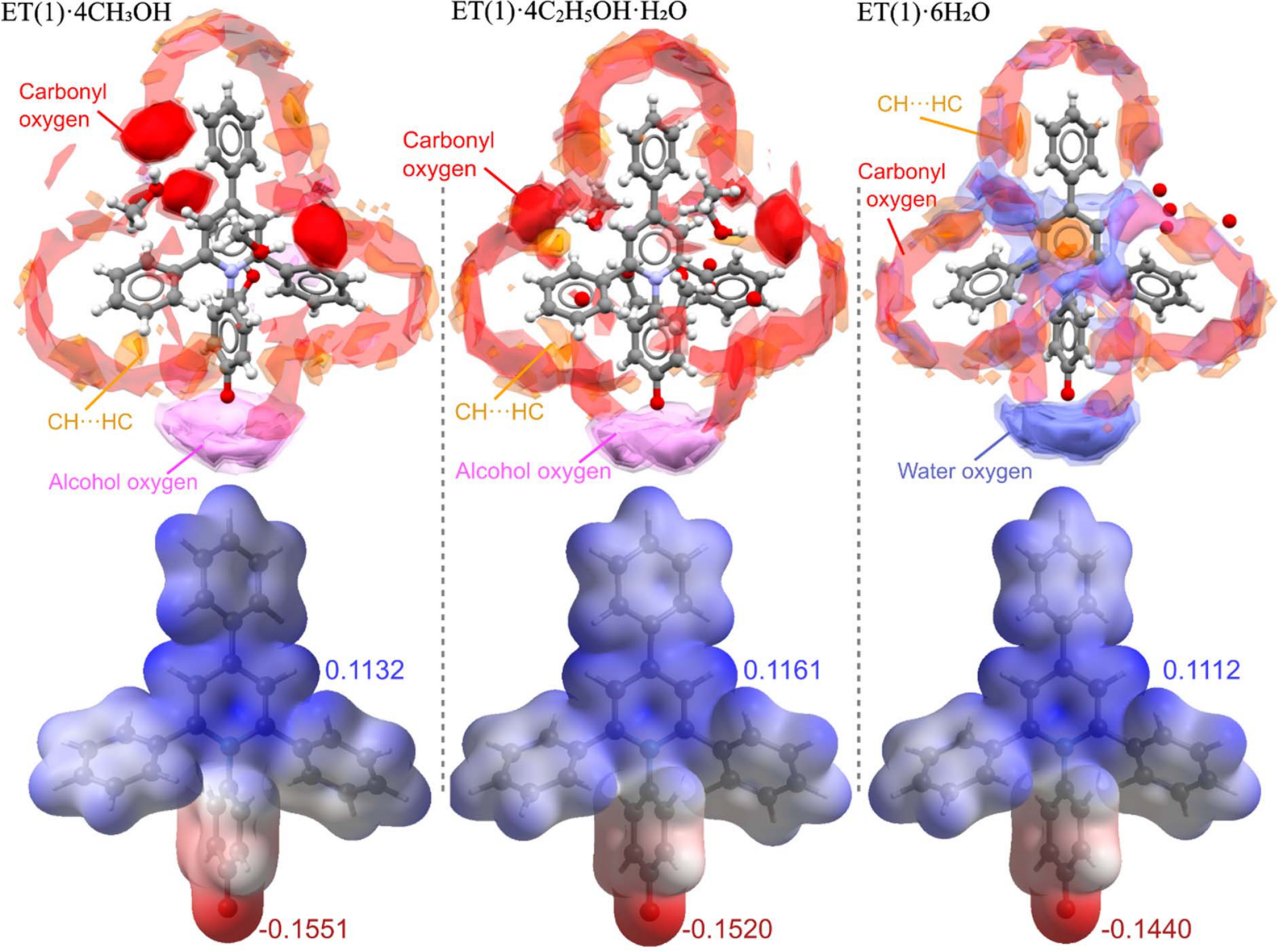
Full interaction maps in ET(1)·4CH_3_OH, ET(1)·4C_2_H_5_OH·H_2_O and ET(1)·6H_2_O solvates and the electrostatic potential plots mapped onto electron density isosurfaces (0.008 e a.u.^−3^) performed in *CrystalExplorer* calculated at the B3LYP/6-(*d*,*p*) level of theory.

Based on the crystallographic data, the ET(1) molecules adopt the most twisted conformation in methanol and are most planar in the water solvate. This result is consistent with the quantum mechanical calculations by Bartkowiak & Lipiński (1998 ▸), suggesting an increase in the molecular dipole moment as angle τ_1_ increases towards 90°. Various possible contributions to the conformational changes include the strain from CH⋯π bonded aggregates, the formation of strong hydrogen-bonds and the intrusion of small solvate molecules into the interstitial spaces between phenyl rings. The close molecular packing in the ET(1)·4CH_3_OH solvate can be attributed to the strong interaction formed between betaine and the solvent molecules, depicted in the interaction mapped in Fig. 7 ▸ (Macrae *et al.*, 2008 ▸). The relationship between the conformational twist of ET(1) and its increasing polarity is corroborated by electrostatic potential calculations performed in *CrystalExplorer* (Spackman *et al.*, 2021 ▸), which indicate the highest values in the methanol solvate. Another characteristic feature of the ET(1) zwitterions is the bending angle measured between the centroids of the core rings. It differs between 180° for the hydrate (where the core rings lie on a twofold axis), 179° for ET(1)·4C_2_H_5_OH·H_2_O and 175.5° in ET(1)·4CH_3_OH; the departure of this angle from 180° can be associated with different interactions on both sites of the molecules, which is confirmed by the increased departure for the high-pressure structures. This angular parameter illustrates the effect of the crystal packing and the surrounding interactions on the molecular dimensions.

## 4. Conclusions

In this study, we explored the solvatochromic behaviour of Reichardt’s dye, ET(1), at high-pressure. Through complementary experimental methods involving high-pressure single-crystal X-ray diffraction and high-pressure UV–Vis spectroscopy, we elucidated the intricate connection between solvatochromism and solvatomorphism for the prototypic representative of these important dyes. The distinctive solvatochromic shifts observed for ET(1) in different solvent environments – methanol, ethanol and acetone – underpin the complex interplay between solvent polarity, hydrogen bonding, molecular conformation and crystal packing. The significant influence of the solvation process on the nucleation of the crystal phases highlights the role of the molecular structure and solute–solvent interactions. The observed piezo-solvatochromic effects contribute to a deeper understanding of molecular chemistry under constrained conditions, shedding light on the stabilization mechanisms of the ET(1) zwitterionic ground state. We have established that (1) Reichardt’s dye, ET(1), favours crystallization in the form of solvates, which show crystal colours similar to their respective solutions; and (2) the intramolecular interactions include, aside from the hydrogen bonds O(1)⋯HO to the solvate molecules and CH⋯π to the neighbouring ET(1) molecules, also the van der Waals contacts to the solvent molecules that penetrate the hollows of the ET(1) molecular surface between the phenyl ring. This latter type of contact is the shortest for the smallest (water and methanol) molecules. It appears particularly important because it affects the inclinations of the phenyls to the core rings, which in turn affects the conjugation of the π electrons, structure and the absorption. The alcohol and water molecules occupy independent sites in the structure, so they interact differently with the ET(1) molecules: ethanol or methanol molecules interact directly with the solvatochromic centres, while the water molecules are weakly associated by dispersive forces, so their effect is marginal. The multiple solvation is not very common – our search of the Cambridge Structural Database (Version 5.45) revealed only 1949 and 475 fourfold methanol or ethanol structures, respectively. What is more, to date, ET(1)·4CH_3_OH and ET(1)·4C_2_H_5_OH·H_2_O are the first structures of organic molecules containing no metal atoms and are solvated by four alcohol molecules. It is remarkable that this multiple solvation occurs for the solvatochromic compounds, where the molecular surfaces form highly selective pockets that are preferential for the solvate molecules with hydro­philic (hydroxyl) and hydro­phobic (aliphatic) residues. These host–guest contacts can be associated with the electrostatic and hydrogen bonds to the zwitterionic sites on one hand, and with the dispersion forces and weak CH⋯C and CH⋯π bonds to the phenyl substituents on the other. The solvatochromic properties can be connected to such a preferential docking of specific solvates, which diminishes the interference of the moisture and the presence of water in the solution.

The implications of this study are far-reaching, particularly in the realm of materials science and nonlinear optoelectronics. The ability to tune the optical properties of Reichardt’s dyes based on pressure opens new avenues for developing advanced photonic materials and pressure-sensitive molecular sensors. Additionally, the insights gained from the solvation mechanism of ET(1) provide basic information about its structure, conformation, interactions and solvation capabilities that are indispensable for the rational design and synthesis of novel molecular dyes with tailored solvatochromic properties for specific applications. The study of solvatochromic shifts under varying pressure conditions opens up new avenues for understanding solvent-mediated effects in molecular systems. The pressure-dependent solvatochromism not only provides a deeper insight into the solute–solvent dynamics, but also paves the way for exploring potential applications in pressure-sensitive molecular sensors and materials science, particularly in the development of advanced photonic materials.

## Supplementary Material

Crystal structure: contains datablock(s) et1_acetone_0_22gpa, et1_meoh_0_57gpa, et1_meoh_1_17gpa, et1_ethanol_0_24gpa, et1_ethanol_0_76gpa. DOI: 10.1107/S2052252524004603/lt5066sup1.cif

Structure factors: contains datablock(s) et1_acetone_0_22gpa. DOI: 10.1107/S2052252524004603/lt5066et1_acetone_0_22gpasup2.hkl

Structure factors: contains datablock(s) et1_meoh_0_57gpa. DOI: 10.1107/S2052252524004603/lt5066et1_meoh_0_57gpasup3.hkl

Structure factors: contains datablock(s) et1_meoh_1_17gpa. DOI: 10.1107/S2052252524004603/lt5066et1_meoh_1_17gpasup4.hkl

Structure factors: contains datablock(s) et1_ethanol_0_24gpa. DOI: 10.1107/S2052252524004603/lt5066et1_ethanol_0_24gpasup5.hkl

Structure factors: contains datablock(s) et1_ethanol_0_76gpa. DOI: 10.1107/S2052252524004603/lt5066et1_ethanol_0_76gpasup6.hkl

Unit-cell projections, magnified CT region in high-pressure absorption spectra and crystallographic data. DOI: 10.1107/S2052252524004603/lt5066sup7.pdf

CCDC references: 2333954, 2333955, 2333956, 2333957, 2333958

## Figures and Tables

**Table 1:** Selected crystallographic data for the ET(1) solvates grown under ambient and high-pressure conditions

	ET(1)·5.78H_2_O	ET(1)·6H_2_O	ET(1)·4CH_3_OH		ET(1)·4CH_3_OH·H_2_O	
Pressure (GPa)	0.0001†	0.22	0.57	1.17	0.24	0.76
Space group	*C*222_1_	*C*222_1_	*P*2_1_/*n*	*P*2_1_/*n*	*R*3*c*	*R*3*c*
Unit-cell parameters						
*a* (Å)	15.005 (9)	15.311 (4)	10.4704 (11)	10.425 (3)	24.062 (9)	23.645 (8)
*b* (Å)	24.356 (4)	23.948 (6)	19.80 (3)	19.26 (7)	24.062 (9)	23.645 (8)
*c* (Å)	7.5097 (9)	7.27 (2)	13.3135 (15)	13.112 (4)	31.259 (8)	30.91 (2)
β (°)	–	–	95.819 (9)	95.70 (3)	–	–
Volume (Å^3^)	2744.5 (17)	2664 (7)	2746 (4)	2620 (10)	15673 (9)	14966 (12)
*Z*/*Z*′	4/0.5	4/0.5	4/1	4/1	18/1	18/1
*D_x_* (g cm^−3^)	1.219	1.275	1.276	1.337	1.134	1.187

† Structural information adapted from WUKYEG determined by Stadnicka *et al.* (2002 ▸).

**Table 2:** Conformation of the ET(1) zwitterions in different solvates under high pressure [Chem scheme1] 
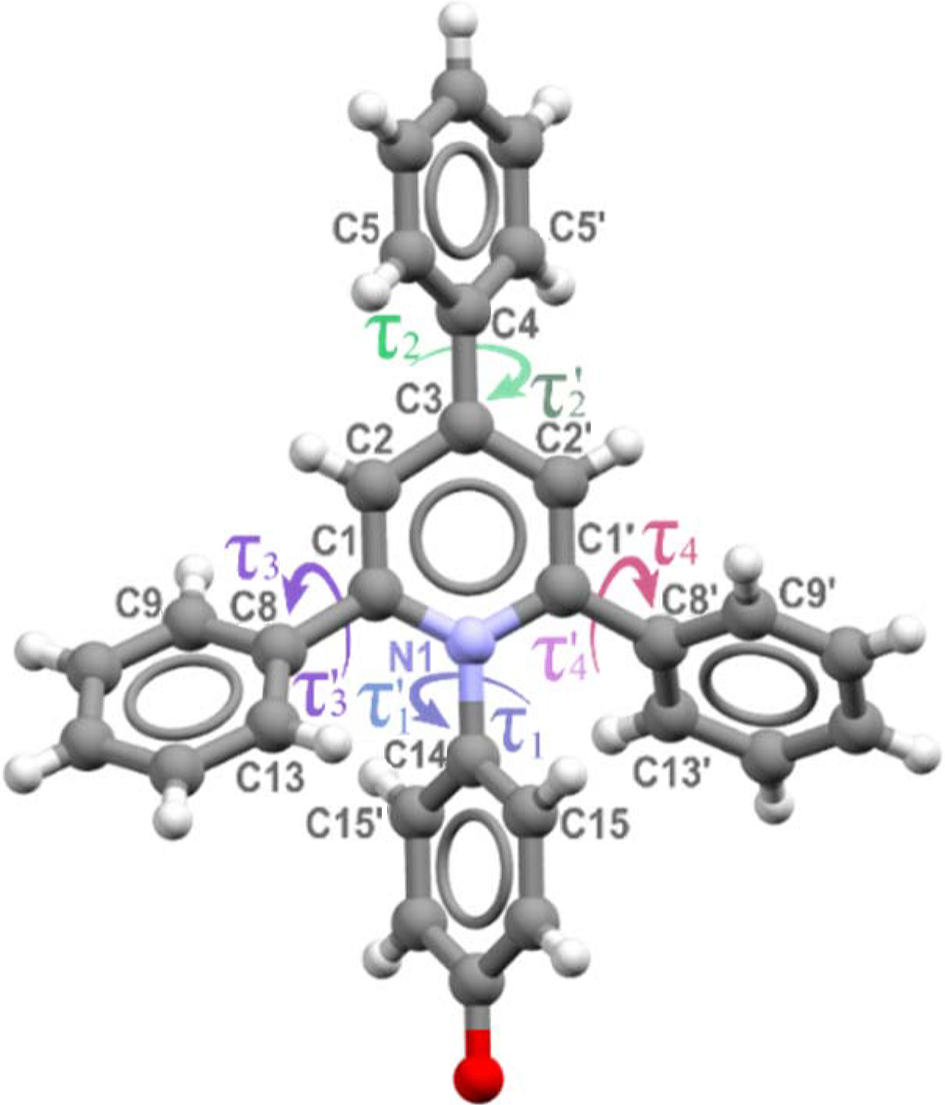

	ET(1)·4CH_3_OH		ET(1)·4C_2_H_5_OH·H_2_O		ET(1)·6H_2_O
	0.57 GPa	1.17 GPa	0.24 GPa	0.76 GPa	0.22 GPa
τ_1_ (°)	75.9 (2)	72.5 (3)	65.6 (3)	48.3 (3)	57.1 (3)
τ_1_′ (°)	71.8 (2)	77.9 (3)	59.2 (3)	74.4 (4)	–
τ_2_ (°)	33.8 (2)	41.0 (2)	20.8 (2)	28.6 (4)	49.2 (3)
τ_2_′ (°)	35.4 (2)	32.6 (2)	34.3 (3)	36.4 (4)	–
τ_3_ (°)	45.8 (2)	48.7 (3)	51.6 (3)	36.4 (2)	40.2 (2)
τ_3_′ (°)	55.0 (2)	45.8 (2)	45.0 (2)	66.0 (3)	57.0 (3)
τ_4_ (°)	55.3 (1)	54.7 (3)	42.1 (2)	41.2 (2)	–
τ_4_′ (°)	58.4 (1)	60.0 (3)	53.1 (3)	53.2 (2)	–
