# Supplementary material for: Structural insight into piezo-solvatochromism of Reichardt’s dye
Source: IUCrJ. 2024 Jun 4;11(Pt 4):528–37. doi: 10.1107/S2052252524004603 (PMC11220889; doi:10.1107/S2052252524004603)
Supplement: Supplementary file 7 [file m-11-00528-sup7.pdf]

IUCrJ

Volume 11 (2024)

Supporting information for article:

Structural insight into piezo-solvatochromism of Reichardt's dye

Szymon Sobczak and Andrzej Katrusiak

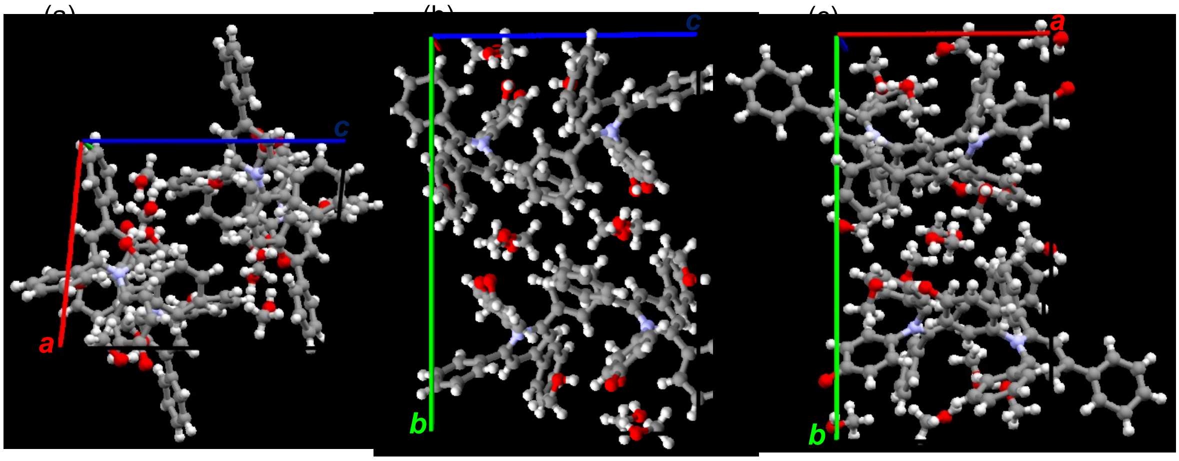

Figure S1 The crystal lattice of ET(1)·4CH_3OH viewed along (a) [010]; (b) [100]; and (c) [001] crystal direction.

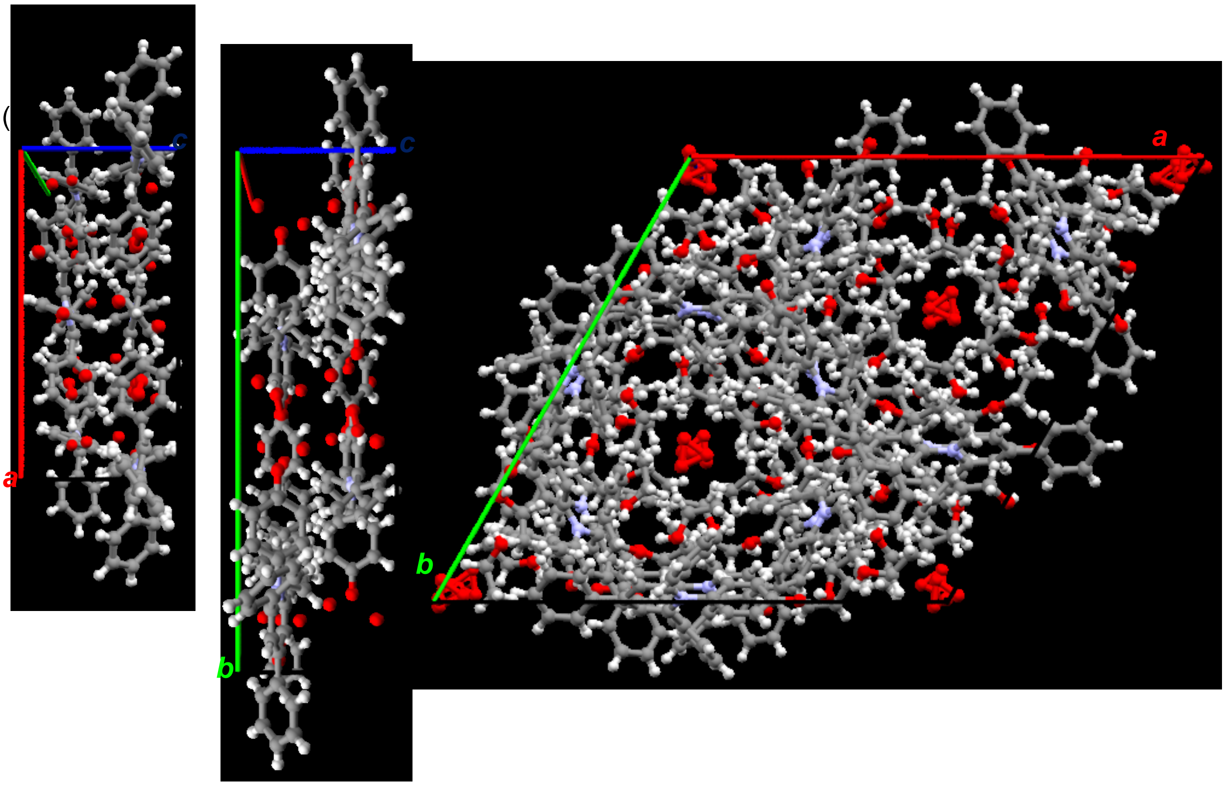

Figure S2 The crystal lattice of ET(1)·4CH_3OH·H_2O viewed along (a) [010]; (b) [100]; and (c) [001] crystal direction.

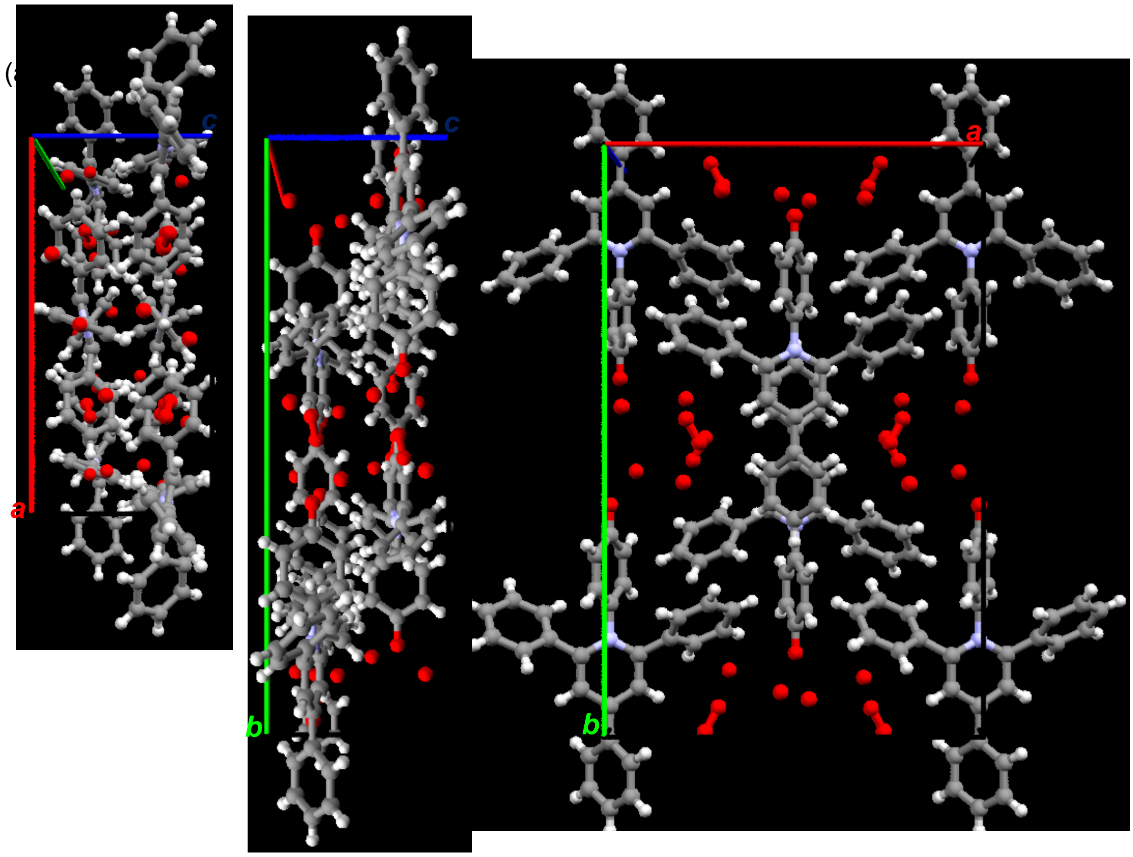

Figure S3 The crystal lattice of ET(1)·$6H_2O$ viewed along (a) [010]; (b) [100]; and (c) [001] crystal direction.

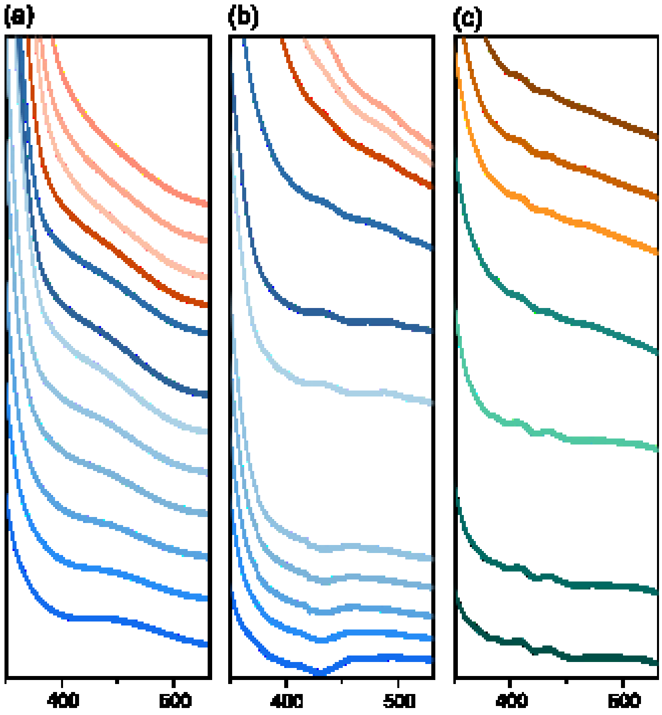

Figure S4 Magnified region of solvent-sensitive solvatochromic bands of the ET(1) solution in (a) methanol; (b) ethanol; and (c) acetone.

Table S1. Crystallographic data of ET(1) in different solvates at room temperature.

Solvate		ET(1)·$6H_2O$	ET(1)·$4CH_3OH$		ET(1)·4C2H5OH·H_2O	
CSD Number		2333954	2333955	2333956	2333957	2333958
Formula weight		527.47	527.63	527.63	599.74	599.74
Pressure (GPa)		0.22	0.57	1.17	0.24	0.76
Temperature (K)		293(2)	293(2)	293(2)	293(2)	293(2)
Crystal system		orthorhombic	monoclinic	monoclinic	trigonal	trigonal
Space group		$C222_1$	$P2_1/n$	$P2_1/n$	$R3c$	$R3c$
Unit cell dimensions (Å,°)	a	15.311(4)	10.4704(11)	24.062(9)	24.062(9)	23.645(8)
	b	23.948(6)	19.80(3)	23.645(8)	24.062(9)	23.645(8)
	c	7.27(2)	13.3135(15)	30.91(2)	31.259(8)	30.91(2)
	α	90	90	90	90	90
	β	90	95.819(9)	90	90	90
	γ	90	90	120	120	120
Volume (Å^3)		2664(9)	2746(4)	2620(9)	14968(16)	15673(12)
Z		4	4	4	18	18
Calculated density (gcm^3)		1.315	1.276	1.276	1.144	1.198
Absorption coefficient (mm^{-1})		0.099	0.085	0.089	0.077	0.08
F(000)		1096	1128	1128	5796	5796
Crystal size (mm)		0.22 × 0.14 × 0.1	0.39 × 0.116 × 0.104	0.33 × 0.13 × 0.05	0.56 × 0.11 × 0.1	0.36 × 0.15 × 0.14
Θ-range for data collection (°)		3.158 to 52.65	4.418 to 57.93	4.46 to 53.602	4.698 to 51.364	6.606 to 52.744
Min/max indices:		-18 ≤ h ≤ 16, -29 ≤ k ≤ 28, -1 ≤ l ≤ 1	-14 ≤ h ≤ 14, -5 ≤ k ≤ 5, -18 ≤ l ≤ 17	-11 ≤ h ≤ 12, -5 ≤ k ≤ 5, -16 ≤ l ≤ 16	-27 ≤ h ≤ 27, -7 ≤ k ≤ 7, -37 ≤ l ≤ 37	-28 ≤ h ≤ 29, -20 ≤ k ≤ 23, -22 ≤ l ≤ 23
Reflections collected		6125	22964	3529	9812	5030
Independent reflections		524 [R_{int} = 0.2170, R_{sigma} = 0.2629]	1643 [R_{int} = 0.1418, R_{sigma} = 0.0879]	1064 [R_{int} = 0.1627, R_{sigma} = 0.2355]	3310 [R_{int} = 0.2013, R_{sigma} = 0.3849]	2002 [R_{int} = 0.2296, R_{sigma} = 0.5157]
Data/restraints/parameters		524/501/150	1643/618/358	1064/864/361	3310/386/360	2002/462/361
Goodness-of-fit on F2		1.27	1.035	1.023	0.974	0.945
Final R_1/wR_2 (I>2δ$_1$)		R_1 = 0.1306, wR_2 = 0.3164	R_1 = 0.0857, wR_2 = 0.2285	R_1 = 0.0795, wR_2 = 0.1889	R_1 = 0.1207, wR_2 = 0.3065	R_1 = 0.1249, wR_2 = 0.2719
R_1/wR^2 (all data)		R_1 = 0.2652, wR_2 = 0.3778	R_1 = 0.1681, wR_2 = 0.2956	R_1 = 0.2272, wR_2 = 0.2803	R_1 = 0.3770, wR_2 = 0.4584	R_1 = 0.3765, wR_2 = 0.4020
Largest diff. peak/hole / e Å^{-3}		0.18/-0.13	0.16/-0.17	0.15/-0.12	0.28/-0.27	0.23/-0.21
